# A Systematic Literature Review of Quality Management Initiatives in Dental Clinics

**DOI:** 10.3390/ijerph182111084

**Published:** 2021-10-21

**Authors:** Emil Lucian Crisan, Bogdan Florin Covaliu, Diana Maria Chis

**Affiliations:** 1 Faculty of Economics and Business Administration, Department of Management, Babes-Bolyai University, 400591 Cluj-Napoca, Romania; emil.crisan@econ.ubbcluj.ro; 2 Faculty of Medicine, Department of Community Medicine, Public Health and Management, Iuliu Hatieganu University of Medicine and Pharmacy Cluj-Napoca, 400337 Cluj-Napoca, Romania; 3 Faculty of Economics and Business Administration, Department of Finance, Babes-Bolyai University, 400591 Cluj-Napoca, Romania; diana.chis@econ.ubbcluj.ro

**Keywords:** quality management initiative, dental clinics, CIMO framework, systematic literature review, oral healthcare quality management

## Abstract

By considering the recently proposed definitions and metrics, oral healthcare quality management (OHQM) emerges as a distinct field in the wider healthcare area. The goal of this paper is to systematically review quality management initiatives (QMIs) implementation by dental clinics. The research methodology approach is a review of 72 sources that have been analyzed using the Context–Intervention–Mechanism–Outcome Framework (CIMO). The analysis identifies five mechanisms that explain how quality management initiatives are implemented by dental clinics. The simplest QMIs implementations are related to (1) overall quality. The next ones, in terms of complexity, are related to (2) patient satisfaction, (3) service quality, (4) internal processes improvement, and (5) business outcomes. This paper is the first attempt to provide a critical review of this topic and represents an important advancement by providing a theoretical framework that explains how quality management is implemented by practitioners in this field. The results can be used by scholars for advancing their studies related to this emerging research area and by healthcare managers in order to better implement their quality management initiatives.

## 1. Introduction

This paper has been developed considering the emergence of oral healthcare quality management (OHQM) as a distinct field of research in the wider medicine quality management area. In the next paragraphs, the theoretical background of this paper is presented, including more topics as the adoption of quality management initiatives (QMIs) in healthcare, the particularities of oral healthcare and QMIs’ implementation in this field, and the main research streams concerning OHQM. We also present our research question within the final paragraph of this section.

The implementation of quality management initiatives in general healthcare organizations has been analyzed in various papers since quality has surpassed in importance the costs of the service [1]. While initially, QMIs were observed in healthcare by considering a more general approach [1], lately, this field has diversified, and more narrow research areas have emerged. The adoption of quality management models, such as the Malcolm Baldrige Quality Award criteria, the European Foundation Quality Management Excellence Model, and the chronic care model, has initially been an important approach, especially for hospitals [2]. In these cases, adoption efforts and QMIs were extensive, and although they affected the whole system, the results of these interventions were limited [2]. Later, the adoption of total quality management (TQM) has been observed as challenging [3], while the six sigma model led to good results related to costs, satisfaction, and resource utilization [4]. In a comparison of the use and the effectiveness of quality management methodologies in surgical healthcare [5], it was revealed that the most used ones are: continuous quality improvement, six sigma, TQM, plan–do–study–act or plan–do–check–act, statistical process or quality control, lean, and lean six sigma. Additionally, in a detailed analysis of lean and six sigma adoption in healthcare, it was presented that six sigma (a detailed and consistent continuous improvement system) has been reported earlier in literature, while lean techniques have been more often found in literature (74,63%), in comparison to lean six sigma (22%), and six sigma (18,15%) [6]. Operations management techniques used in the healthcare industry, such as VSM (visual stream mapping) and standardization of work and visual management, are also recognized as widely used techniques [6]. More narrow analyses deal with the adoption of specific quality management tools in the healthcare industry. A simple Kano model is recognized as very hard to be used in healthcare since there are many variations regarding customer needs and preferences concerning different types of care provided by healthcare providers [7]. The use of SERVQUAL in healthcare services for assessing their quality has also been tested, revealing the importance of promptness of response received by patients, cleanliness and hygiene, and empathy of doctors and employees as main areas of quality perceived by patients [8].

Oral health is recognized as an important determinant for overall health and well-being, and from a statistical point of view, it can be associated with physical, mental, and general health, energy levels, work limitation, depression, and appetite [9]. It is estimated that dental diseases accounted worldwide in 2015 for USD 356.80 billion as direct costs (dental expenditure), while indirect costs associated with these diseases were estimated at USD 187.61 billion (productivity losses) [10]. Oral healthcare is different from general care, being characterized by: regular and asymptomatically participation of patients, primarily surgical nature, associations with pain and anxiety, and primarily cosmetic and secondarily disease treatment nature [11]. Moreover, dental practitioners pay their own wages by the number of patients and interventions they make and are involved in commercial activities, with the dental patient adopting customer rather than patient attitudes [11]. Though these obvious differences exist, OHQM has adopted in time practices previously used in general medicine quality management. The use of Donabedian’s structure, process, outcome system approach on quality management [11,12,13,14,15], and the use of the quality dimensions proposed by the Institute of Medicine (IoM) (safety, effectiveness, timeliness, patient-centeredness, efficiency, and equity quality dimensions) [12,13,16], similar to the dimensions proposed by Donabedian [17,18] and used by Campbell and Tickle [11], have also been identified for OHQM.

Much of the recent research concerning OHQM is focused on the development of concepts and defining quality. Much of the research is associated with defining quality in this field. It is recognized that quality in oral healthcare is poorly defined in comparison to quality in general medicine [11,12,13,14], and that the lack of a generally accepted definition and measurement of oral healthcare quality blocks its development [11,15,16]. A working definition for quality of oral healthcare has been proposed, including seven domains (patient safety, timeliness, patient-centeredness, equitability, efficiency, effectiveness, and accessibility) and 30 items [19]. Other conceptual contributions for OHQM target the introduction of adequate sets of measures [12,13,15], as well as the establishment of specific goals relevant only for OHQM (which should be generally accepted by practicians, thus providing a unified definition of quality management) [11,12,13]. A systematic literature review concerning the metrics used in OHQM reveals that they are mainly related to patients’ satisfaction, 9 out of 11 studies presenting evidence for this patient-centered quality management approach, while the rest are related to self-assessment of practice made by a dentist or a manager [12]. Efficiency (costs related aspects), and equity are poorly considered.

Secondly, besides these conceptual papers, there are papers and sources that bring evidence that quality management in oral healthcare is transposed into regional and national standards of initial education and continuing professional development of dental professionals [20]. OHQM is also presented as an activity governed by the state, multinational bodies such as the European Union, or professional associations [14], which establish policies such as the Quality in Dentistry policy proposed by the FDI World Dental Federation [21].

Finally, there are papers that analyze the context and the results of quality management practices adoption in healthcare. These results are contradictory. Though these quality management practices have been proven to positively influence healthcare organization performance [22], it is found that leaders/managers of healthcare organizations are not necessarily well trained or even the right persons for launching such quality management initiatives [23], and the adoption itself has failed in many organizations [24]. Contextual factors such as leadership, organizational culture, data infrastructure and information systems, high experience in QMI implementation [25], but also human resources involvement and their knowledge [26] are recognized as important factors affecting the success of quality management implementation in healthcare. The lack of a systemic approach and the adoption of rather microsystemic improvements have been regarded as sources for the lack of success in the case of QMIs in healthcare [27].

After considering the existing OHQM data available in the scientific literature, it is obvious that research in this field is at its beginnings, being mostly concerned with defining quality and establishing metrics. We take this one step further with this systematic literature review and answer the following question: how do dental clinics implement QMIs, as reported by the literature? In order to answer this question, we analyze empirical papers that present QMIs in dental clinics by considering a system design approach—the CIMO Framework proposed by Denyer et al. [28]. This approach is capable of explaining when and why (context—C), how (intervention—I), and with what results (outcome—O) dental clinics implement specific QMIs. Moreover, this framework targets the identification of explanatory mechanisms regarding how different dental clinics combine C, I, and O. The main result of such an analysis is a theoretical framework that aggregates and explains the QMIs already implemented in practice, this framework being an important input for further advancements in the field.

## 2. Research Design

**Figure 1:**
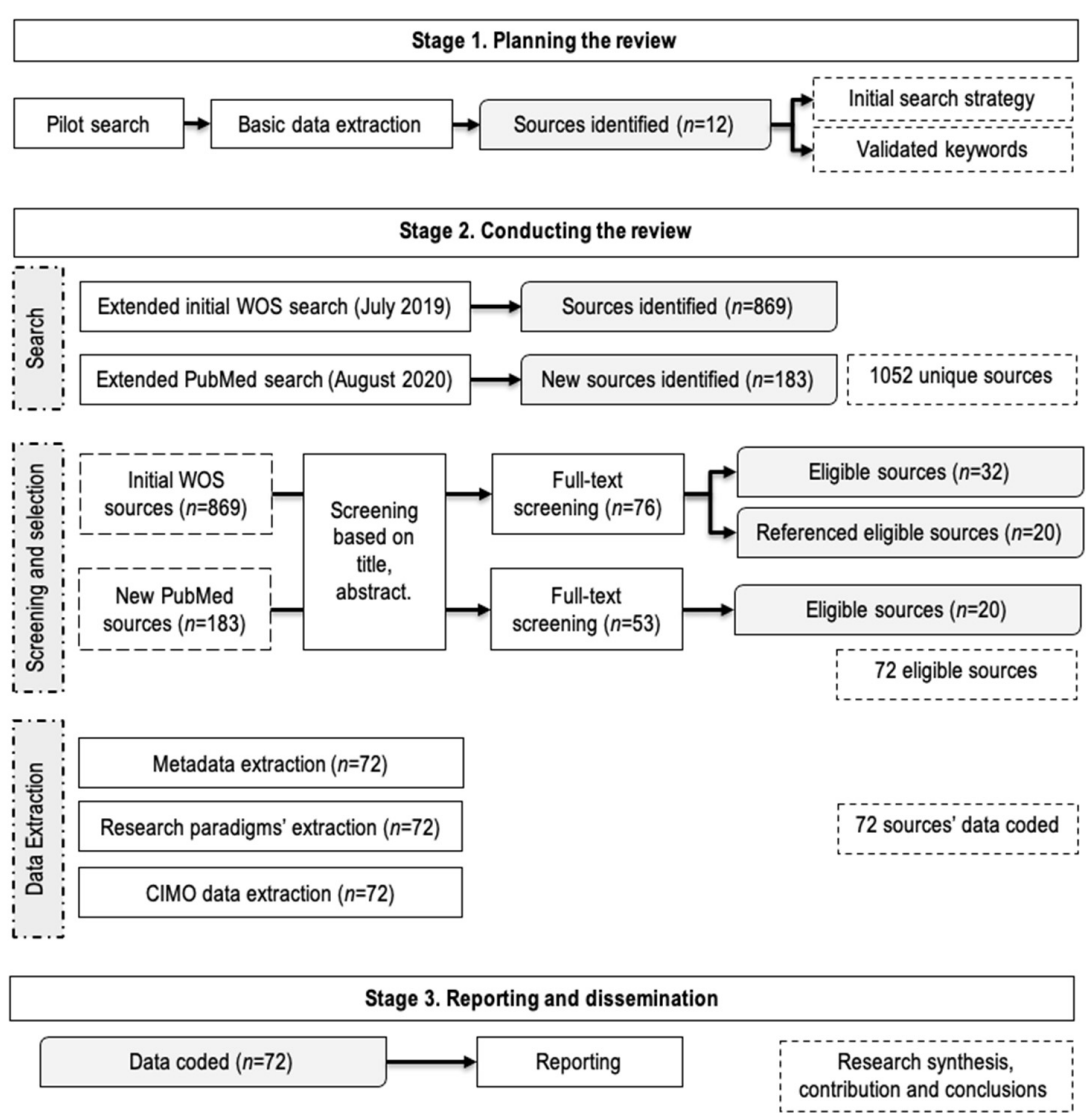
Research process.

The literature review follows the methodology proposed by Tranfield et al. [29], this methodology being appreciated by medical and quality management researchers due to its transparent and replicative nature [6,7,30]. This methodology is commonly used within management literature, being similar to the one detailed within the PRISMA declaration for medical research [31], since it has been developed considering previous methodologies developed in medical science [29]. Figure 1 describes the procedure we have followed, including the activities we undertook during each stage.

### 2.1. Planning the Review

The first step was to establish the goal of the review, and that all articles and case studies covering dental clinics QMIs, published at all times, should be included in the review. Later, a pilot search that led to the identification of 12 articles was conducted, this search being used to establish the search strategy and to identify the search terms.

### 2.2. Performing the Review

Two extended searches were performed in July 2019 (in Web of Science—WoS database) and August 2020 (PubMed database). The initial WoS search included multiple terms in TOPIC (title, abstract, keywords), generating the following number of articles: “dentistry” “customer satisfaction”—7, “dentistry” “quality assessment”—112, ”dentistry” “quality improvement”—43, “dentistry” “quality assurance”—82, “dentistry” “quality management”—11, “dental” “customer satisfaction”—20, “dental” “quality improvement”—142, “dental” “quality assessment”—359, “dental” “quality assurance”—273, “dental” “quality management”—43 articles. This search led to 1092 sources, out of which 223 duplicates were removed, bringing the total to 869 unique sources. A similar PubMed search was performed, all our search terms, with the exception of “customer satisfaction”, being registered as medical subject headings terms. This search led to the identification of 1316 sources, out of which 186 duplicates were removed, bringing the total to 1130 unique sources. These sources were later compared with those from WoS sources, and after excluding duplicates, 183 unique studies were added to the WoS ones. Given the nature of the topic, which belongs both to the management and the medicine field, similar to other reviews, we have considered that the two searches in WoS and PubMed will provide the main relevant sources for our analysis. WoS was chosen due to its comprehensiveness, as it includes a wide range of academic sources in the management field [32], while PubMed is the mostly used database for medical literature [33]. Moreover, relevant articles referenced in the previously selected articles have been included in our analysis in order to also cover grey literature, as further described.

The screening of the 1052 sources’ titles and abstracts was conducted using three inclusion criteria: (I1) the articles should present cases related to dental clinics; (I2) the topics addressed in these articles are related to QMIs; (I3) all the papers are written in English. Furthermore, three exclusion criteria were considered: (E1) papers that focus only on a specific part (pathology) of dental treatment (e.g., implant); (E2) papers that focus only on a specific protocol or method related to dental treatment (e.g., radiology); (E3) papers related to national strategies. Each source was analyzed by two of the authors and, in case of doubt, the article was fully read and discussed until a common agreement was reached. The result of screening was 129 eligible sources from both searches. Three reviewers examined the content of these sources, with two reviewers evaluating each source. The same inclusion and exclusion criteria were used, 32 sources related to the WoS search and 20 related to the PubMed search being validated. After extracting the data from sources, 20 new articles, cited by initial sources, were also validated. Therefore, 72 sources were used in this systematic literature review (see Table 1).

All researchers were involved in extracting information from all eligible sources using an online data collection template. This template included metadata fields such as: authors, year of publication, title, publication title, item type (journal article, book chapter, conference paper, report), methodology type (qualitative, quantitative), methodology research methods, OHQM focus (primary or secondary), QMI geographical area, and relevant cited sources.

Data extraction and analysis has been performed following the CIMO framework, which typically produces design propositions that encapsulate patterns of context, interventions, and outcomes to describe the examined phenomenon—in this case, QMIs’ implemented by dental clinics. In management science, it has been previously used to capture how organizational and inter-organizational phenomena occur [105,106]. In comparison to the PICOS criteria which are used to identify components of clinical evidence for systematic reviews in evidence based medicine [107], CIMO is mostly used for organizational design-oriented research synthesis [28]. CIMO structure provided the theoretical framework for our approach to coding, which was mostly deductive: we identified a list of codes to reflect the contexts, interventions, and outcomes of QMIs’ implementation and used these existing constructs to make sense of our data by identifying the explanatory mechanisms. For QMIs’ context, two fields have been considered: one for the nature (research/clinical purpose) of the QMI and one for the QMIs’ triggers/expected benefits. Interventions were extracted in a specific field, while for outcomes, four relevant fields have been used: one for the outcomes (similar to triggers/expected benefits identified for context), and two for the nature of the outcome (one for real outcomes versus ideas or recommendations and one for qualitative versus quantitative outcomes).

## 3. Results

### 3.1. Descriptive Analysis

The 72 selected sources were published between 1974 and 2020. Almost 50% entered the literature after 2010 (*n* = 35), with a peak of six articles in 2017. Furthermore, 69 of the sources are journal articles, 2 are conference papers, and 1 is a report. Concerning the country of origin, a large number of sources examine dental facilities in the USA (*n* = 28), and a significant number of sources (*n* = 11) looked at the UK. The majority (*n* = 57) of studies refer to dental clinics in Europe and North America. Additionally, 65 sources have quality management as their primary goal, the others having quality management as a secondary one.

The most common research methods were quantitative methods, observed in 64 of sources, and implied statistical analysis of questionnaires results (45 sources) and quantitative observations from patients’ applications, dental care files, integrated electronic health records, list of patient complaints, informed consents, etc. (21 sources). The least commonly used methods were the qualitative methods (15 sources), such as summarizing, categorizing, or interpreting interviews responses (five sources), focus group discussions (three sources), and other qualitative methodologies (eight sources).

### 3.2. CIMO Results

#### 3.2.1. Context

As stated by Denyer et al. [28], the context is related to the external and internal environment factors that impact behavioral change. Considering the nature of the intervention, QMIs’ context has been divided into research driven (29 studies), where the intervention was associated with a research project/initiative, and clinical driven (43 studies), where the intervention was initiated internally by the clinic without an exclusive research goal.

Five main categories regarding QMIs adoption triggers/expected benefits have emerged (see Table 2). The most common category of triggers of QMIs adoption is patient satisfaction, also mentioned as a more patient-centered oral healthcare, or the improvement of the patient–provider relationship (41 studies). This result confirms the increasing importance placed by the patient [70], and that patient satisfaction generally has been accepted as an important element of OHQM [34]. The second most common category of expected benefits refers to the improvement of professional practice and organizational activities (33 studies), including the improvement of professional practice and the overall practice of clinics, reshaping dentist practice patterns, increasing awareness and the level of knowledge among dentists, improving the quality of the work environment, minimizing the time-consuming and stressful patient-search process, managing the quality of products and services delivered to the customer, confidentiality and security, increased opportunities for reusing electronic data for quality assurance and research, etc. [52,84,88,96]. The third category, recognized in 32 studies, refers to more general triggers for dental organizations: improving the overall quality of care and health outcomes, improving access and reducing disparities in oral healthcare, etc. [39,53,90]. The fourth category of dental clinics expected benefits related to QMIs adoption, mentioned in 23 studies, is enhancing service quality of dental care, involving the improvement of the quality of delivered healthcare services, increasing the overall supply of dental services available, and encouraging utilization of dental health services, commitment to provide a high-quality service, achieving and ensuring good service quality to meet or exceed, delivering a more effective oral health service to residents, improving the service quality of care for homeless and vulnerably housed people, etc. [36,44,60,81]. Finally, the least mentioned category of expected benefits, appearing in only 13 studies, is related to the improvement of dental clinics business outcomes: clinic efficiency, revenue, profit, attracting new patients, enhancing dental care service providers performance and gaining customer preferences, cost containment/savings, financial stability, cost-effectiveness of the new service delivery model, etc. [37,53,103].

#### 3.2.2. Intervention

The most common category of interventions (see Table 2), applied in the majority of the analyzed cases (37 studies), is the evaluation of patients’ opinions/perceptions (satisfaction, complaints, factors influencing the access to oral healthcare services, criteria they use to choose a dentist, the communication they have with dentists, etc.), followed by dental care providers and other staff opinions (satisfaction, elements regarding the organizational environment, the use of information systems, etc.). The evaluated studies use various instruments (questionnaires, indicators, etc.), mainly in order to improve patient satisfaction and the delivery of high-quality dental services [44,67,83]. Another important and very common category of interventions, mentioned by 35 studies, are the ones that propose the implementation and/or the assessment of quality improvement programs, such as: implementation of a pay-for-performance incentive program for medical personnel; developing a new model of dental record; examining the effectiveness of a quality improvement and management program consisting of a set of quality indicators for external and internal dimensions; using quality improvement methods to implement an early childhood oral health program; a program to reduce the number of patients’ failed appointments; the development and implementation of an integrated model of care using oral health practitioners and tele-dentistry; assessing the implementation of an educational program related to dental care, etc. [14,53,97,102]. In contrast, the least common category of interventions is the adoption of technology and digitization instruments. Considered only by eight studies, this type of intervention included: the development of a prioritization system; a screening website that improves access to care for patients and assists in the matching of patients and students; use of electronic health records; evaluating the effectiveness of a pre-play communication instrument; shifting from a time-based to an item-based fee-paying system in order to improve patient satisfaction; introduction of an automated confirmation system of appointments, etc. [38,48,51,96].

#### 3.2.3. Outcomes

While reviewing the outcomes for each case, we have observed that the nature of the outcomes varies from real outcomes (24/72 of sources). While real outcomes present themselves in the form of real improvements/changes related to QMIs, the nature of most outcomes are exposed in the form of proposals, including ideas and/or recommendations as a result of QMIs’ adoption. The majority of studies had presented only proposals as a result of their intervention (48/72), while seven studies (7/72) present both real results and propositions.

By considering the qualitative (e.g., improved documentation, better care) and quantitative (results which could be measured, e.g., a two-fold increase in diagnostic and treatment services capacity) nature of the presented outcomes and the research-driven or clinical-driven nature of the QMIs, we observed two patterns. Research-driven studies had qualitative outcomes (25/29 cases), and their results were mostly in the form of ideas and recommendations (27/29), while clinical-driven sources had more quantitative outcomes (17/43 compared with 5/29), and the proportion of these studies that had real outcomes was larger than the ones that had research as their main purpose (21/43 compared to 3/29).

Another analysis we made regarding the outcomes focused on the dimension to which they refer. In this analysis, the previously identified categories for triggers/expected benefits in the context section were used, and each outcome was matched with a corresponding trigger. For most studies included in our analysis, outcomes are part of multiple categories (only 10/72 studies have reported outcomes included into a single category). Overall quality and access to oral healthcare related outcomes (proposals and real outcomes) were reported in 47 cases. Outcomes related to patient satisfaction, patient-centered oral healthcare, and patient–provider relationship are present in 40/72 sources. Outcomes concerning oral healthcare service quality were revealed in 29/72 sources. Outcomes related to the improved professional practice and organizational activities have been identified in 35/72 sources. Finally, business outcomes were present in only 12/72 sources.

## 4. Mechanisms and Discussion

The main contribution provided by our systematic literature review, performed through the use of CIMO framework, is the highlight of the explanatory mechanisms for the phenomenon of QMIs. Regarding QMIs’ adoption (as presented by the 72 analyzed sources), these can be explained considering two perspectives: one related to the triggers of the interventions (in this case, two mechanisms being observed: research-driven and clinical-driven QMIs) and another that focuses on the nature of the intervention (in this case, five design propositions or mechanisms being observed, as further described).

### 4.1. Mechanisms

By considering QMIs’ nature, we have identified QMIs that cover different areas regarding quality. These areas are similar to the five-stages framework, which explains small and medium enterprises’ approaches of quality management proposed by Yang [108]: product quality (product related quality control and process inspection practices), process quality (process standardization practices), system quality (quality management system such as ISO practices), total quality (much emphasis is given to customer focus and a quality culture across the organization), and business quality (quality becomes a matter of business strategy, being related to strategic management, human resource management, or business performance). In our case, the mechanisms cover five areas: internal processes, patient satisfaction, service quality, overall quality, and business outcomes.

Internal processes improvement is the second most frequently encountered mechanism for QMIs (24 cases). The context ranges from the desire to improve the professional practice and the overall practice of dental providers, to reshape dentist practice patterns, and improve the level of knowledge among dentists. The most encountered category of interventions was the implementation and assessment of quality improvement projects, programs, and methods, due to the fact that this mechanism is more focused and specialized on clinical activities and management practices, and it included specific and unique initiatives that improve their practices [14,45,64]. The majority of outcomes in this category are qualitative and imply ideas and recommendations for improving dental quality management and professional practices. An important part of outcomes is represented by qualitative and quantitative real outcomes, such as improved documentation [35], reduced waiting list [48], improved work environment [89], enhancement of interdisciplinary collaboration [66], and reduction in number of missed appointments [97].

Patient satisfaction is the most frequently encountered mechanism (25 cases). In this case, the context categories involve the need to increase patient satisfaction, more patient-centered oral health care, and the improvement of the patient–provider relationship. The interventions involve three categories of initiatives, and some clinics propose a combination of more initiatives [82,86]: evaluation of patients’ satisfaction using various instruments (questionnaires, indicators, etc.), the use of quality improvement programs, and technology and digitization instruments. In this case, the majority of outcomes are qualitative, patient–customer-focused proposals, mainly emphasizing means to improve patient satisfaction and patient–provider relationship. Nevertheless, some studies present real quantitative and qualitative patient satisfaction improvements [63,65].

Service quality mechanism is encountered in 11 cases. The context, in this case, refers to some particular triggers: the improvement in the quality of delivered healthcare services, the increase in the overall supply of available dental services, and increasing and encouraging utilization of dental health services. The main intervention here is the evaluation of patients’ opinions, using questionnaires and other instruments, considering the importance of patients’ expectations in achieving and ensuring good service quality. The majority of outcomes are represented by qualitative outcomes in the form of proposals to enhance dental service quality. Additionally, there are some qualitative and quantitative real outcomes focused on services improvements [51].

Overall quality is the third most frequently encountered mechanism (13 cases). The context refers to some expected benefits/triggers that focus on the improvement of the overall quality of care and health outcomes, the improved access to oral healthcare, and the reduction in disparities in oral healthcare. The main intervention in this category implies quality improvement programs [55,93,103], and only a few cases refer to the evaluation of patients and dental care providers’ opinions using various instruments (questionnaires, indicators, etc.) regarding the quality of care [70,71]. As opposed to the above mechanisms, in this category (overall quality) the majority of outcomes are quantitative real outcomes, such as oral healthcare quality and patients’ care improvements [50,102].

Finally, the business outcomes represent the least frequent mechanism, recognized in nine cases. The context, in terms of the business expected benefits, vary from the desire to improve clinical efficiency, to increased revenues and profits, cost savings, financial stability, and increased number of patients. Although the most encountered type of intervention is represented by unique and specific quality improvement programs, an important role is also attributed to technology and digitization tools such as introducing an automated appointment confirmation system [38]. Similar to the previous mechanism, the most encountered category of outcomes is quantitative real outcomes, mainly increased number of patients [37], increased efficiency due to broken appointments rates reduction [38,97], costs savings [97], financial stability, and increased revenues [103].

### 4.2. Discussion

QMIs adoption by dental clinics is performed in the context of research projects or is clinically driven. By considering the 72 sources included in the current review, the existence of these two approaches, with research-driven interventions in 29 studies and clinical-driven interventions in 43 studies, explains the variation observed especially when we consider real outcomes and proposed outcomes that derive from these interventions. However, the proposed mechanisms that explain QMIs’ adoption are similar to maturity models presented by the literature [108]. Based on our analysis, it can be observed that QMIs can have narrow internal focus, such as improvement of processes, but are mainly externally driven (patient satisfaction and service improvements). Larger focuses, such as overall quality and business outcome mechanisms, have been also identified. The five mechanisms explain the evolutionary nature of quality adoption in any organization, which usually starts from simple internal processes improvements and later develops a customer focus (patient satisfaction and service quality in our case), followed by a quality management system focus, and finally, a business impact focus, similar to the self-assessment tool for SMEs created by Sturkenboom et al. [109]. The main reasons for not passing to the more evolved stages are the lack of knowledge and resources [110], or in the case of dental clinics, the lack of a generally accepted definition and measurement tools for oral healthcare quality management [11,15,16].

While comparing the initial triggers and expected benefits when adopting QMIs, it can be observed that the number of outcomes exceeds the number of triggers, especially while considering the proposals associated with QMIs. Supplementary outcomes were identified in relation to the initial proposed context (based on our counting a 14% increase was observed), which can suggest either insufficient planning of the intervention, or external factors driving to other results related to specific QMIs.

Additionally, considering the two areas—research and clinical-driven cases—the most encountered category of clinical-driven studies outcomes is qualitative and involves proposals for future overall quality of dental care improvements, while the research-driven cases mainly provide proposals for patient satisfaction and patient–provider relationship improvements, as well as proposals for improving the overall quality of dental care. Moreover, besides the service quality mechanism, the rest of our mechanisms were observed mostly in clinical-driven studies, probably because the clinical real interests are mainly related to internal processes, patient satisfaction, overall quality improvements, and obtaining better business outcomes. Almost a third of interventions had a research purpose only, without impacting the actual quality of the system. However, the majority of studies were initiated internally based on real needs.

## 5. Conclusions

We have reviewed 72 papers in order to observe how quality management initiatives are implemented by dental clinics. Five design propositions or mechanisms were observed, ranging from overall quality to patient satisfaction, service quality, internal processes improvement, and business outcomes. The main focus of quality management in this field is related to patients’ satisfaction, followed by process improvements, a balance between internal and external-driven quality management initiatives being observed. It is obvious that more systemic approaches are required [14], business outcomes being targeted at a low level. Dental clinics’ organizational capabilities, as quality management is also defined, should be considered towards the technical capabilities as an important area of oral healthcare. Although about twenty years have passed, the four core properties of successful quality-improvement work proposed by Ferlie and Shortell [1] (leadership, culture, teams, and technologies) are still insufficiently implemented in the field of dental care.

Considering the five mechanisms identified in our study, it has become clear that dental clinics managers should perform a detailed analysis on the fitness of a specific QMI for their organizational context. Depending on internal needs, proper QMIs should be selected. Similar to the initiatives implemented in healthcare in general (ex. [24]), the implementation of different quality management initiatives should be well planned and communicated across the organization, otherwise the results of the initiatives could be different than the ones initially targeted. The development of more complex quality management initiatives that have, as a goal, the improvement of business outcomes should also be considered by dental clinic managers, since patient satisfaction and process improvements are important as long as they are linked to more customers and increased financial benefits.

## Figures and Tables

**Table 1:** Sources included in the review and main CIMO details.

Authors and Year	Research/Clinical Purpose	Mechanism
[34]	Research	Overall quality
[35]	Clinical	Internal processes improvement
[36]	Research	Patient satisfaction; Service quality
[37]	Clinical	Business outcomes
[38]	Clinical	Business outcomes
[39]	Research	Patient satisfaction
[40]	Research	Internal processes improvement
[41]	Research	Service quality
[42]	Research	Overall Quality
[43]	Clinical	Patient satisfaction
[44]	Research	Service quality
[45]	Clinical	Internal processes improvement
[46]	Clinical	Service quality
[47]	Clinical	Overall quality
[48]	Clinical	Internal processes improvement
[49]	Research	Patient satisfaction; Service quality
[50]	Clinical	Overall quality
[51]	Clinical	Patient satisfaction; Service quality
[52]	Clinical	Internal processes improvement
[53]	Clinical	Business outcomes
[54]	Clinical	Overall quality
[55]	Clinical	Overall quality
[56]	Research	Patient satisfaction
[57]	Research	Internal processes improvement
[58]	Research	Patient satisfaction
[59]	Research	Internal processes improvement; Business outcomes
[60]	Research	Patient satisfaction; Service quality
[61]	Research	Patient satisfaction
[62]	Clinical	Service quality
[63]	Clinical	Patient satisfaction
[64]	Clinical	Internal processes improvement
[65]	Clinical	Patient satisfaction
[66]	Clinical	Internal processes improvement
[67]	Clinical	Patient satisfaction
[68]	Clinical	Overall quality
[69]	Clinical	Internal processes improvement
[70]	Research	Overall quality
[71]	Research	Overall quality
[14]	Research	Internal processes improvement
[72]	Research	Patient satisfaction
[73]	Clinical	Patient satisfaction
[74]	Clinical	Internal processes improvement; Business outcomes
[75]	Clinical	Patient satisfaction
[76]	Clinical	Internal processes improvement
[77]	Research	Service quality
[78]	Research	Service quality; Business outcomes
[79]	Clinical	Service quality
[80]	Research	Patient satisfaction
[81]	Clinical	Patient satisfaction
[82]	Clinical	Patient satisfaction
[83]	Research	Patient satisfaction
[84]	Research	Internal processes improvement; Business outcomes
[85]	Clinical	Internal processes improvement
[86]	Clinical	Patient satisfaction
[87]	Clinical	Patient satisfaction
[88]	Clinical	Internal processes improvement
[89]	Clinical	Internal processes improvement
[90]	Clinical	Overall quality
[91]	Research	Internal processes improvement
[92]	Research	Internal processes improvement
[93]	Clinical	Overall quality
[94]	Clinical	Internal processes improvement
[95]	Clinical	Patient satisfaction
[96]	Research	Internal processes improvement
[97]	Clinical	Internal processes improvement; Business outcomes
[98]	Research	Internal processes improvement
[99]	Clinical	Patient satisfaction
[100]	Research	Patient satisfaction
[101]	Research	Patient satisfaction
[102]	Clinical	Overall quality
[103]	Clinical	Overall quality; Business outcomes
[104]	Clinical	Internal processes improvement

**Table 2:** Context, intervention and outcomes summary.

Dimension	Coded Aspects	Results/Cases
**Context**	QMI nature (research/clinical purpose)	Research-driven QMIs—29/72 Clinical-driven QMIs—43/72
	QMIs’ triggers/expected benefits	Patient satisfaction—41/72 Improvement of professional practice and organizational activities—33/72 General triggers—32/72 Enhancing service quality—23/72 Improving business outcomes—13/72
**Intervention**	Open coding	Evaluation of patients’, dental care providers and other staff satisfaction opinions etc.—37/72 Implementation and/or the assessment of different quality improvement programs—35/72 The adoption of technology and digitization instruments—8/72
**Outcomes**	Nature of the outcome (real outcomes versus ideas or recommendations)	Real outcomes—24/72 Proposals—48/72 Both—7/72
	Nature of the outcome (qualitative versus quantitative)	Research-driven studies—qualitative outcomes—25/29 Research-driven studies—quantitative outcomes—5/29 Clinical-driven QMIs—qualitative outcomes—28/43 Clinical-driven QMIs—quantitative outcomes—17/43
